# Spatiotemporal parameters and kinematics differ between race stages in trail running—a field study

**DOI:** 10.3389/fspor.2024.1406824

**Published:** 2024-06-24

**Authors:** Matteo Genitrini, Julian Fritz, Thomas Stöggl, Hermann Schwameder

**Affiliations:** ^1^ Department of Sport and Exercise Science, University of Salzburg, Hallein-Rif, Austria; ^2^ Athlete Science Department, Adidas AG, Herzogenaurach, Germany; ^3^ Red Bull Athlete Performance Center, Thalgau, Austria

**Keywords:** trail running, race stage, kinematics, spatiotemporal parameters, performance, technique

## Abstract

**Introduction:**

Trail running is an emerging discipline with relatively few studies performed in ecological conditions. The aim of this work was to investigate if and how spatiotemporal parameters (STP) and kinematics differ between initial and final stage of a field trial.

**Methods:**

Twenty trail runners (10 F, 10 M) were recruited and ran a solo 9.1 km trial. During the test, participants wore a GPS watch and an IMU-based motion capture system. Running speed, elapsed time, STP and kinematics were compared between initial and final stage, separately for uphill (UH) and downhill (DH) sections.

**Results:**

Running speed decreased in the final stage (p<0.05). Total test time was more correlated to the time elapsed in UH sections. In the final stage and in both UH and DH sections, contact time and duty factor increased, whilst stride length and flight time decreased (p<0.05). In the final stage, ankle joint was more dorsiflexed in stance and swing phases in UH sections and stance phase only in DH sections (p<0.05). In the final stage, knee joint was less extended in swing phase in UH and DH sections, as well as less extended in stance in UH sections (p<0.05). In the final stage, hip joint was less flexed in the swing phase in UH and DH sections (p<0.05). In the final stage, forward trunk lean was higher across the entire gait cycle in in UH sections (p<0.05). Trunk contralateral axial rotation was lower, in DH sections (p<0.05).

**Discussion:**

During the final stage, results indicate a less efficient propulsion phase, in both UH and DH sections. In UH sections, results suggest lower energy generation at the ankle joint. In DH sections, results suggest that the kinematics of swing leg may play a role in sub-optimizing propulsion phase. This study demonstrates how, in UH and DH sections, similar changes in spatiotemporal parameters can be elicited by dissimilar changes in running kinematics. To optimize performance in trail running, coaches and practitioners are advised to work on different (incline-specific) aspects of running technique.

## 1 Introduction

Trail running is an increasingly popular endurance discipline, defined as any foot race taking place in a natural environment (eg. mountains, forests, deserts etc.). Not more than 20–25% of the race length may be paved or asphalted, with athletes spending most of the time on a trail, dirt road or a single track. There are no limits as for race length and elevation gain, therefore race distance may range between ∼5 km up to several hundreds [1]. One of the most demanding factors in trail running is the incline, with possibly long and technically challenging uphill (UH) and downhill (DH) sections. Advances in wearable technology enabled trail running research outside the laboratory, thus allowing to gain insights about biomechanics and physiology in ecological conditions. When focusing on specific aspects such as fatigue-induced adaptations in late race stages, a restricted number of investigations monitored running biomechanics and/or physiology *during* a trail running trial, comparing the final stage to the initial one. In a 3-laps field test (3,175 m/lap), Townshend et al. [2] reported a decrease in stride length (SL) in the final stage, as well as a decrease in oxygen uptake. Similarly, in a 2-laps field test (3,524 m/lap), Born et al. [3] reported a decrease in oxygen uptake, respiratory exchange ration, minute ventilation and oxygen pulse in the final stage. The authors also reported an increase in heart rate and breathing frequency, compared to early race stages. Björklund et al. [4] reported that the greatest time loss, in short trail running trials, occurs in UH sections, when comparing initial and final stages.

Technique has been defined as “a specific sequence of movements or parts of movements in solving movement tasks in sports situations” [5]. When considering kinematics, examples of technique-related variables include peak flexion/extension joint angles, range of motion and joint angles at specific time points of a gait cycle, to name a few. With respect to performance, in running competitions the goal is usually to attain the highest average running speed as possible to cover a given distance. Since running speed is the product of SL*stride frequency (SF), and SF being 1/2 (contact time (CT) + flight time (FT)), it is apparent how any change in one or more of such parameters would directly influence running speed. Therefore, spatiotemporal parameters (STP) represent in running the performance-related variables. Previous articles have pointed out how, in many cases, a distinction between variables that characterize technique and variables that characterize performance is not made [6]: the author highlighted how oftentimes coaches are informed about performance-related variables, but not about how they may positively impact them by acting on specific technique-related variables. With this in mind, kinematics and STP may be valuable tools to better understand the technique - performance nexus in trail running.

Comparing different training sessions in similar disciplines as overground level running, Van Oeveren et al. [7] reported that higher speeds are associated with higher SF, SL, FT, as well as to lower CT and duty factor (DF), compared to lower speeds. Considering a decrease in running speed associated with acute fatigue, Apte et al. [8] reported an almost identical trend in STP. Genitrini et al. [9] observed a similar trend in STP in trail running as well, when comparing two groups of faster and slower trail runners. Therefore, it may be concluded that the trend in performance-related variables (i.e., STP) is substantially the same when running at different speeds, regardless the cause that elicited a difference in running speed (be task instructions, fatigue or performance level).

Conversely, such considerations do not seem to apply to technique-related variables (i.e., kinematics). In fact, Preece et al. [10] investigated non-fatigued athletes instructed to run at different speeds, reporting differences in hip and knee angles at initial contact across speeds. Conversely, investigating speed decrease associated with fatigue, Apte et al. [8] found no differences at the hip and knee joints, but reported lower plantar flexion at initial contact. Lussiana et al. [11] emphasized the importance of individual running patterns characterized by different kinematics, distinguishing between aerial and terrestrial patterns. It has been suggested that this could have relevant implications for training and rehabilitation purposes [12]. With respect to trail running and comparing faster to slower athletes, Genitrini et al. [9] reported higher knee and hip joint peak flexion during swing and larger trunk rotation. To summarize, adaptations in technique-related variables at different running speeds appear more situation-specific, compared to adaptations in performance-related variables.

To the best of our knowledge, no study investigated the effects of race stage on both kinematics and STP in trail running yet. These findings would convey valuable insights about how, in late race stages, technique-related variables influence performance-related variables. In particular, coaches and practitioners may target specific (and possibly different) aspects of technique in UH and DH sections, thus positively impacting running speed in late race stages.

It was hypothesized that running speed would decrease from the initial to the final stage of a trail run. Also, it was hypothesized that the concurrent changes in STP (performance-related variables) would resemble those observed comparing faster and slower trail runners [9]. In particular, we hypothesized that SF, SL and FT would decrease and CT and DF would increase, in the final stage. As for technique-related variables, we hypothesized that kinematics would differ between initial and final stage, and also in a dissimilar fashion between UH and DH sections. In this regard, we adopted an explorative approach, given that adaptations in technique-related variables appear to be situation-specific.

## 2 Materials and methods

### 2.1 Participants

Twenty subjects (10 M, 10 F) were recruited from local trail running associations (age [years]: 32.8±8.3 M, 33.4±8.1 F; body height [cm]: 177.2±6.0 M, 166.3±6.9 F; body mass [kg]: 71.9±5.8 M, 61.6±6.9 F, experience in trail running [years]: 3.3±1.5 M, 4.1±1.2 F). All participants were amateur athletes habitually competing at regional to national level. The study was approved by the ethical committee of the University of Salzburg (GZ-10/2022), in conformity to the declaration of Helsinki.

Inclusion criteria were:

• Between 18 and 50 years old
• At least 1 year experience in trail running
• Minimum training frequency 2 times per week
• Minimum weekly training volume 30 km
• No injuries in the previous 3 months prior to the study

### 2.2 Protocol

**Figure 1:**
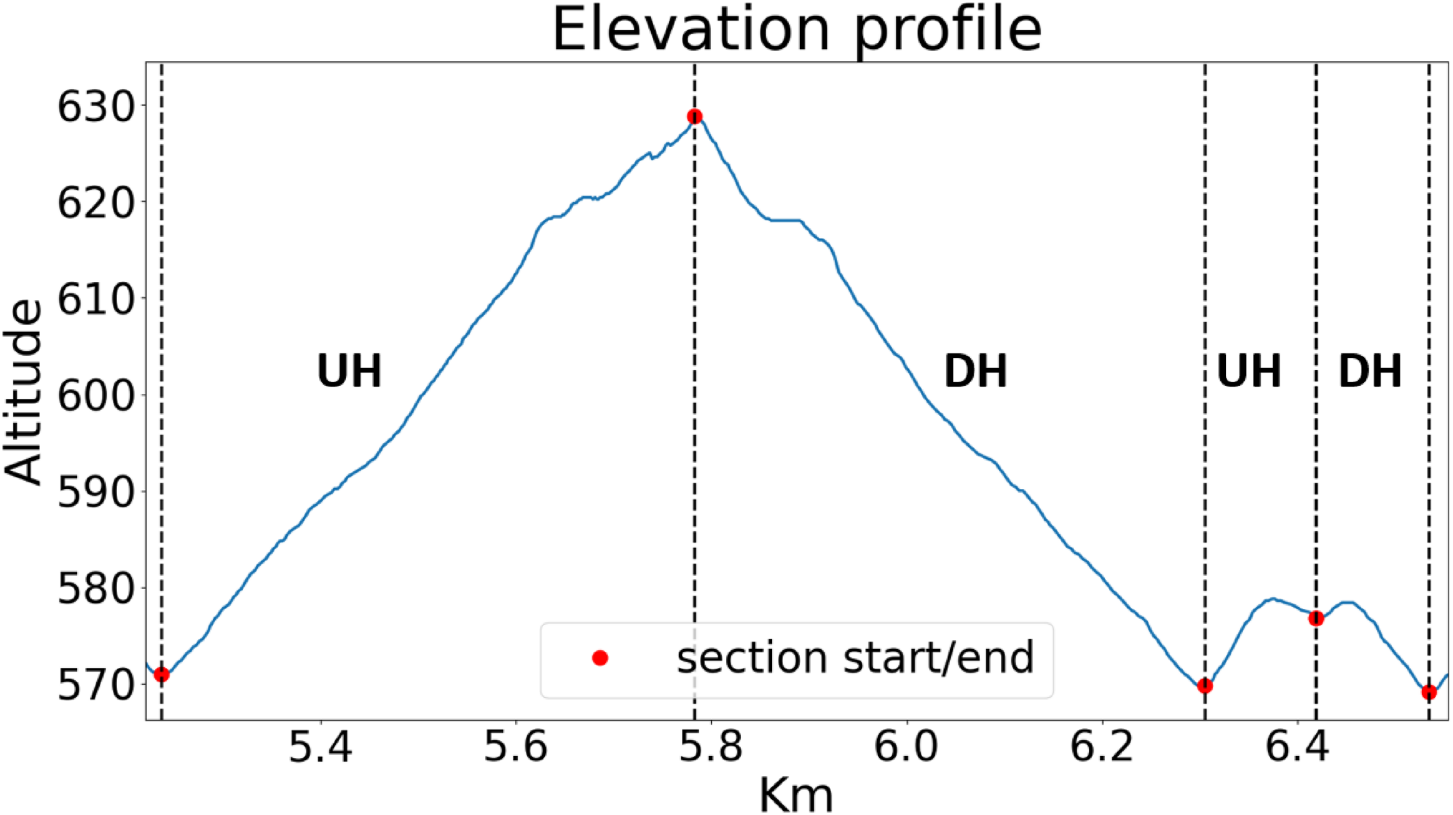
Elevation profile of trail running test lap; a full test consisted of seven repetitions. For the analysis, the 7th lap was excluded due to the possible presence of end spurts.

After providing their informed consent, anthropometric data including body height, body mass and length of relevant body segments were recorded. On a separate day, participants completed a ∼9.1 km trail running time trial consisting of 7 laps of the same 1.3 km route (see Figure 1). Each lap presented an ascent of 60 m, resulting in 420 m of positive and negative elevation gain across the entire trial. Before the test, participants were accompanied during a complete lap of the running route to familiarize themselves with the test environment, followed by a self-selected warm up, consisting of level and incline running, as well as static and dynamic stretching exercises. Participants were instructed to complete the test in the shortest time as possible, without jeopardizing their safety in any instant of the trial. During the test, time and positional data, STP and full body kinematics were recorded.

All subjects were tested between April–August. The temperature during tests was 23.2 ± 3.7∘C, with sunny or cloudy weather conditions. No tests were performed with rain. Terrain was dirt road consisting of a stable combination of gravel and pine needles, on which athletes could safely and confidently run with no risk of falls. Sporadically, a few traits of asphalt were present as well. Due to the presence of trees, most of the route was in the shadow, thus avoiding that the athletes continuously ran under the sun.

### 2.3 Materials

During the trail running test, participants wore a GPS watch (Garmin Forerunner 935) and a full body motion capture system (Xsens Link, Xsens Technologies BV, Enschede, The Netherlands). The latter consisted of 15 inertial measurement units (IMUs, model MTx, size 36×24.5×10 mm, mass 10 g, sampling frequency 240 Hz). IMU sensors were located on: head, shoulders (2×), arms (2×), forearms (2×), thighs (2×), legs (2×), feet (2×), sternum and pelvis. Previous studies validated such system against gold standard marker-based methods, reporting reliable and consistent results for tasks such as running and changes of directions on both asphalt and irregular surfaces [13–16]. All subjects wore the equipment approximately 30 min before the beginning of the trial, thus having enough time to become accustomed with the suit and the wearables. Those subjects who asked for it, could run with their own watch as well, thus one at each wrist.

### 2.4 Data analysis

Since trail running trials are often characterized by speed decreasing across the entire task (i.e., positive pacing) with a end spurt in the very last stages [17], the 7th lap was excluded, as possibly not representative of the overall course. Subsequently, laps 1–3 were classified as initial stage, whilst laps 4–6 where classified as final stage. Within each lap, two UH and two DH sections were identified (see Figure 1). The whole data analysis was performed in a Python environment, after exporting joint angle and sensors raw acceleration data.

#### 2.4.1 Spatiotemporal parameters

Accuracy of GPS technology in outdoor environments is a well-established topic. With respect to distance, previous validation works reported an average underestimation error of 6% in forest environments, for the watch model used in this study [18]. To overcome this potential bias in the calculation of STP, we used as a reference the distance reported on a widely used website for outdoor activities: https://www.outdooractive.com/. For subsequent calculations, such reference distance was used for all subjects, as they all ran the same route. With respect to the reference, the error in the distances measured with the GPS watch in the present study was 6.61±2.20%, thus comparable with Gilgen-Ammann et al. [18].

For the calculation of STP, linear acceleration for the foot IMU sensors was used. By means of a previously validated algorithm [19], acceleration peaks corresponding to initial contact and toe off were identified. SF of each gait cycle was calculated as the opposite of the time elapsed between two consecutive ipsilateral initial contacts. CT of each gait cycle was calculated as the time elapsed between initial contact and toe off. FT of each gait cycle was calculated as 0.5*(1/SF)-CT; it was assumed that contralateral initial contact occurred at 50% of each gait cycle. DF for each gait cycle was calculated as 100*(SF*CT). With respect to SL, this parameter was computed dividing running speed by SF. In each section, running speed was calculated dividing the reference distance (standardized for all subjects) by elapsed time. Elapsed time was calculated as the difference in the time stamps at the beginning at the end of each section. Each section started/ended in a peak/valley (see Figure 1). Therefore, relevant time stamps were identified as local maxima/minima in the altitude data from the GPS watch. Such procedure was adopted so not to rely on the distance data returned by the GPS watch, thus avoiding introducing any bias in the calculations.

#### 2.4.2 Joint angles

With respect to joint angles, data of ankle, knee, hip and trunk were processed with the software MVN Analyze^©^ (Xsens Link, Xsens Technologies BV, Enschede, The Netherlands) in HD mode and No-Level scenario, thus yielding joint angle trajectories as recommended for biomechanical applications [16]. Finally, data were exported.

#### 2.4.3 Walking gait cycles

As running is characterized by a flight phase where neither foot is in contact with the ground, a DF lower than 50% indicates running, whilst values above such threshold indicate walking. In trail running it is not unusual to switch between running and fast walking, especially in UH sections. Nonetheless, since the present study focuses on running biomechanics, walking gait cycles were excluded, as well as subjects walking more than certain thresholds. Based on DF, walking gait cycles were identified.

Those subjects walking more than 10% of total gait cycles were excluded. It means to exclude those subjects who, on average, interrupted their running more frequently than once every 10 strides. We suggest that less than 10 consecutive strides from running start is an interval too short to reliably assess running technique. Those subjects walking 2.5–10% of total gait cycles, were retained if the number of walking gait cycles in the initial and final stage did not differ by more than 10%. It means that those athletes who stopped running every 10–40 strides were retained only if they walked to a very similar extent between race stages. In fact, we suggest it would be suboptimal to compare biomechanics of running bouts of very different length, between race stages. Therefore, we discarded those subjects who tended to walk remarkably more often in a certain race stage. Those subjects walking less than 2.5% of total gait cycles were retained.

### 2.5 Data reduction and statistics

Data reduction was performed by averaging data (or summing, for the elapsed time), separately for UH and DH sections, and each race stage. In this way, for each participant, STP, joint angles and elapsed time were calculated in UH as well as in DH sections in the initial and final stage.

With respect to STP, running speed and elapsed time, separately for UH and DH sections, the assumption of normality was checked by means of the Shapiro-Wilk test, revealing normal distribution of all parameters in all conditions. Therefore, differences between initial and final stage in STP, running speed and elapsed time were assessed via paired sample t-test. Separately for UH and DH sections, differences in joint angles kinematics between race stages were assessed with Statistical Parametric Mapping [20], paired t-test. Moreover, to provide information about which sections are more strongly correlated to overall performance, the linear regression between total test time and time elapsed in UH and DH sections is reported as well. Level of significance was set to alpha = 0.05.

## 3 Results

**Figure 2:**
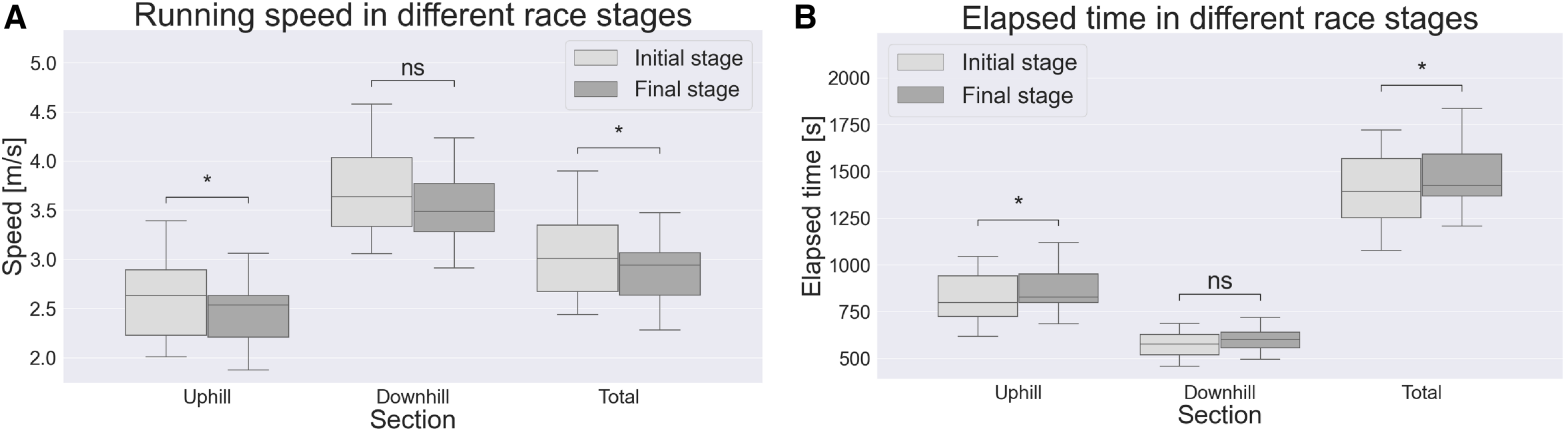
Running speed (**A**) and elapsed time (**B**) comparison between race stages; ∗, p<0.05; ns, not significant.

One female athlete was not able to complete the test. Based on exclusion criteria (more than 10% of gait cycles walking, or 2.5–10% with >10% difference between race stages), data from 6 subject were discarded, resulting in a sample size of 13 (5 F). With respect to performance, average running speed across the entire route was significantly lower (p<0.05) in the final stage. In particular, this resulted from a significantly lower running speed in UH sections (p<0.05) and a trend towards lower running speed in DH sections (p=0.06), see Figure 2A. Elapsed time was higher in UH sections, compared to DH sections, see Figure 2B.

**Figure 3:**
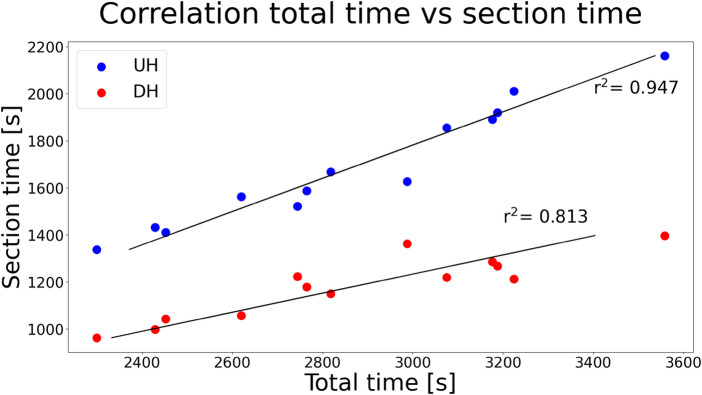
Linear regression between total time and time spent in each UH or DH section. In both cases, p<0.05.

The regression model returned a significant correlation of total test time with elapsed time in both UH and DH sections, see Figure 3.

**Figure 4:**
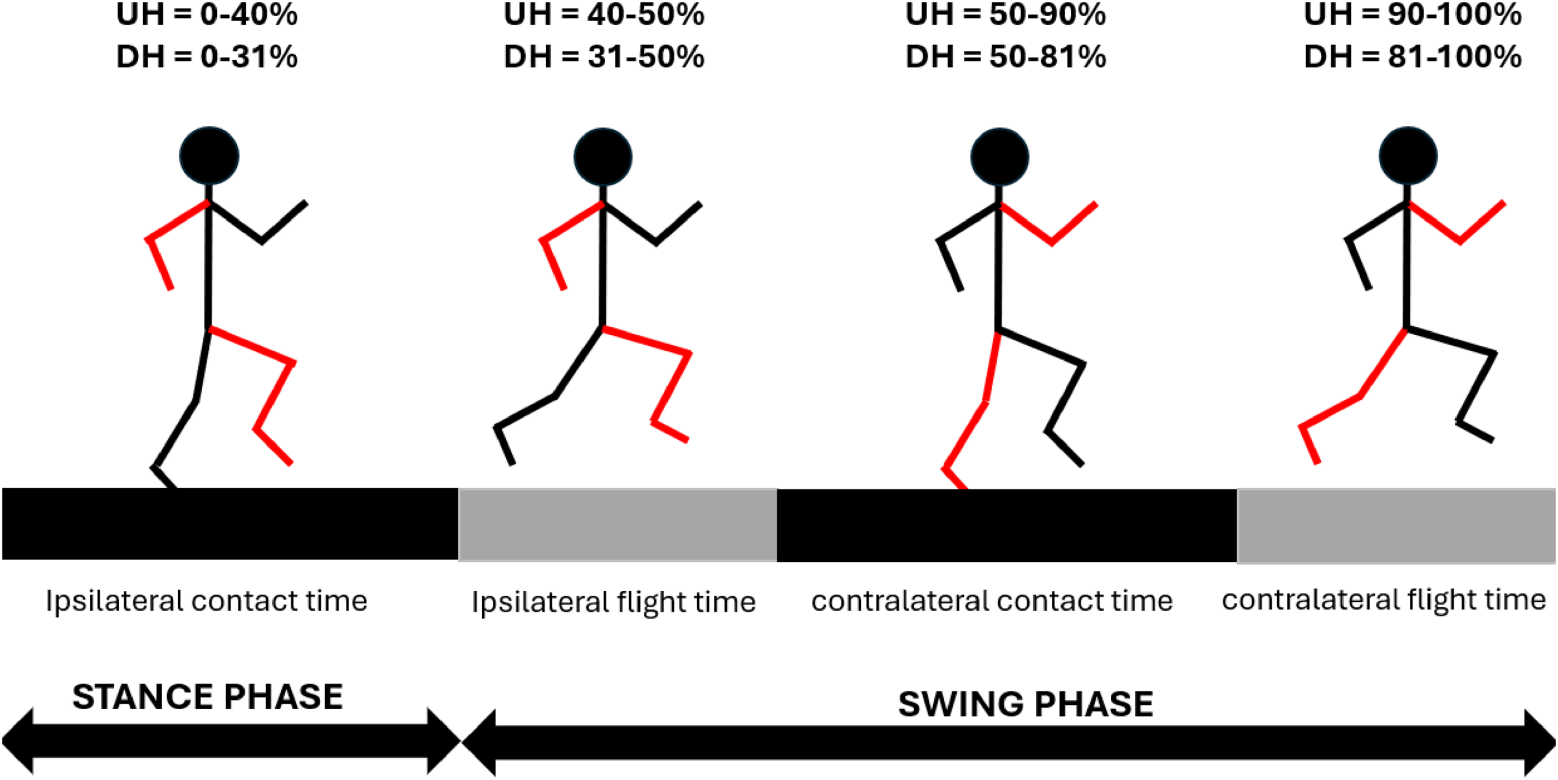
Schematic representation of gait phases. Values are reported separately for uphill (UH) and downhill (DH) sections. Within each section, since the difference between race stages was ∼1%, values were averaged for clarity.

**Figure 5:**
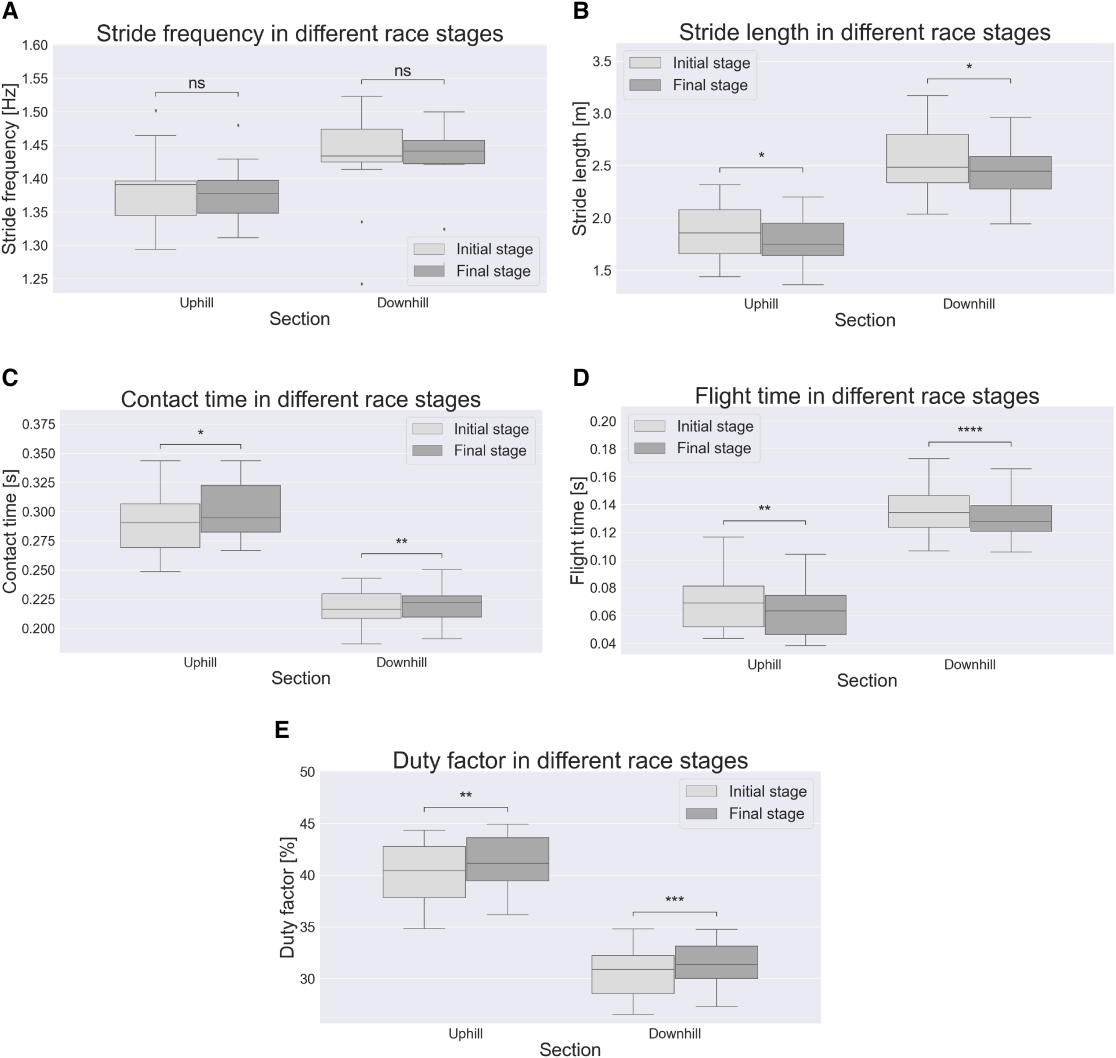
Spatiotemporal parameters by terrain and race stage. ∗, (p<0.05); ∗∗, (p<0.01); ∗∗∗, (p<0.001); ∗∗∗∗, (p<0.0001); ns, not significant difference between race stages.

As for STP, in Figure 4 a schematic representation of gait phases is provided. SF did not differ between race stages (p>0.05), see Figure 5A. SL was lower in the final stage in both UH and DH sections (p<0.05), see Figure 5B. CT was higher in the final stage in both UH and DH sections (p<0.05), see Figure 5C. FT was lower in the final stage in both UH and DH sections (p<0.05), see Figure 5D. DF was higher in the final stage in both UH and DH sections (p<0.05), see Figure 5E.

### 3.1 Kinematics

**Figure 6:**
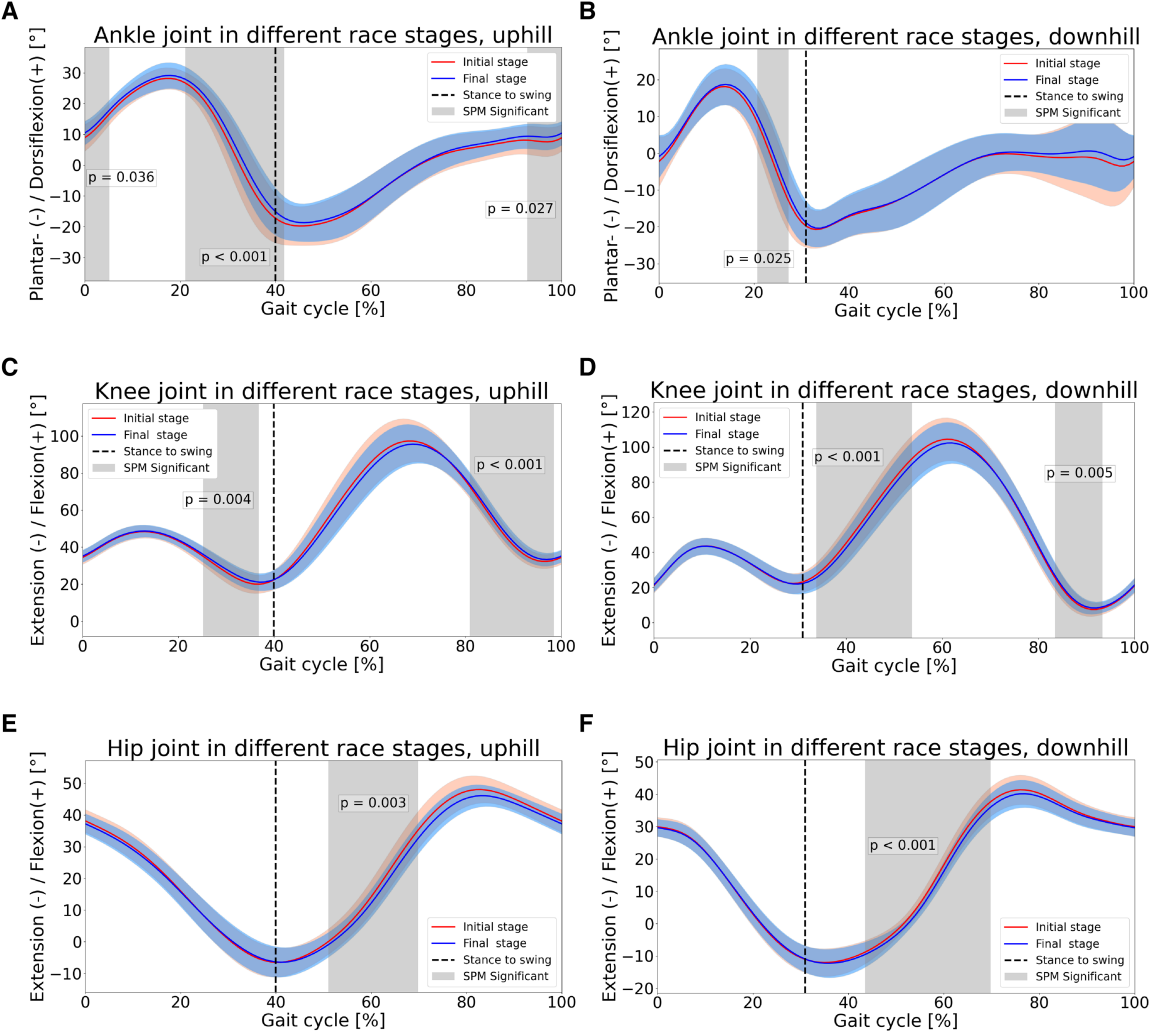
Lower body joint angles in different race stages. First row: ankle; second row: knee; third row: hip.

**Figure 7:**
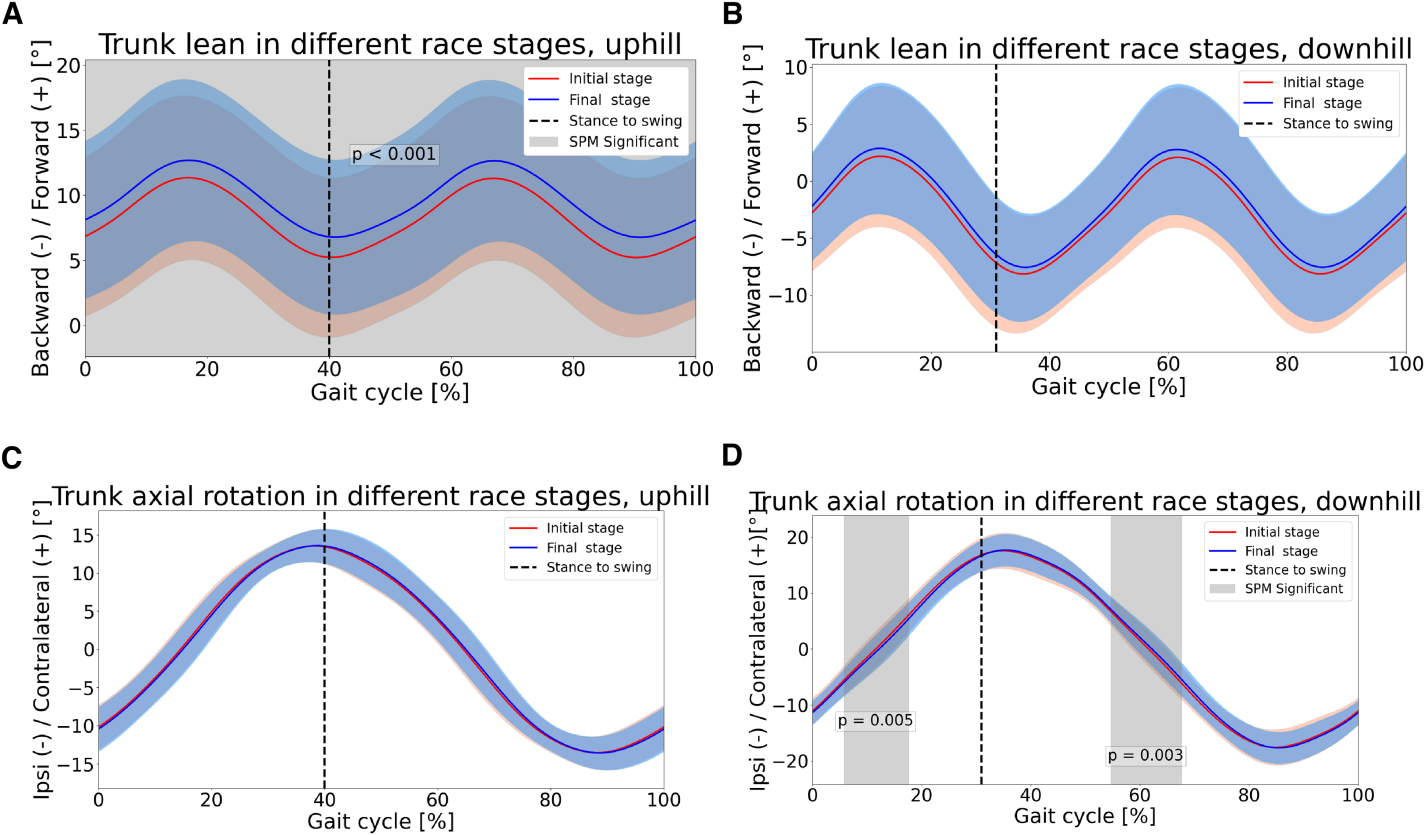
Upper body joint angles in different race stages. First row:trunk lean; second row: trunk axial rotation.

Significant differences were found between race stages with respect to kinematics (Figures 6–7). As for the ankle joint, in both UH and DH sections, dorsiflexion was higher in the final stage, during stance phase (p<0.05), see Figures 6A,B. In UH sections, higher ankle dorsiflexion was found immediately prior to foot strike as well (p<0.05), see Figure 6A. With respect to knee joint, in UH sections the athletes expressed higher flexion in late stance phase (p<0.05), in the final stage. In swing phase instead, for both UH and DH sections the range of motion (ROM) was lower in the final stage, as a result of similar peak flexion and lower peak extension, see Figures 6C,D. With respect to hip joint, significant differences were found during swing phase in both UH and DH sections during flexion sub-phase (p<0.05). In particular, athletes expressed lower flexion in the final stage, see Figures 6E,F. With respect to trunk lean athletes expressed higher forward tilt across the entire gait cycle in UH sections, during the last race stage (p<0.05). Conversely, no differences were found in DH sections, see Figures 7A,B. With respect to trunk axial rotation, in DH sections athletes expressed lower contralateral rotation during mid stance phase, in the final stage (p<0.05). No differences were found in UH sections, see Figures 7C,D.

## 4 Discussion

The hypothesis of overall speed decrease in the final stage was confirmed. In particular, such decrease in performance is explained by lower running speed in UH sections and a trend towards decreased running speed in DH sections. These findings are consistent with previous studies focusing on short trail running trials. In particular, Björklund et al. [4] reported that the greatest time loss in a 7 km trail running trial occurred in UH sections, like in the present study (Figure 2B). Similarly, Ehrström et al. [21] reported finish time to be more strongly correlated to split times in UH than in DH sections, in a 27 km trail running trial. Also in this regard, our results are consistent (Figure 3). Nonetheless, present findings conflict with those reported for longer races (i.e., >100 km). In particular, Genitrini et al. [22] found a greater decrease in performance (investigated as relative speed) in DH sections, when comparing initial and final stage.

### 4.1 Uphill

As for UH sections, the hypothesis of lower SL in the final stage was confirmed. As SF did not change, lower running speed in the final stage is uniquely caused by lower SL, consistently with the findings of Townshend et al. [2], who reported that running speed depends more on SL rather than on SF. The hypothesis of longer CT in the final stage was confirmed as well. In this regard, previous works reported a negative relationship between running speed and CT [7], as well as higher CT in fatigued conditions [8]. In UH conditions, muscles must perform primarily positive net mechanical work to increase the potential energy of the center of mass (COM), during propulsion phase. Previous studies showed that, in UH conditions, the main contributor to positive net mechanical work is hip, followed by ankle and knee [23]. Despite differences in hip joint kinematics were found in swing phase only (Figure 6E), in the final stage we found lower ankle plantar flexion and lower knee extension during propulsion phase (Figures 6A,C), as well as during late swing phase, immediately prior to foot strike. Such differences result into lower ROM during propulsion phase, due to lower ankle plantar flexion and knee extension, in the final stage. Previous investigations reported that higher running speed in UH running is associated to higher ROM and higher energy generation, at the ankle joint [24]. Given this kinematic-kinetic link and the lower ROM found at the ankle in the present study, our results suggest lower energy generation at the ankle joint during propulsion phase, in the final stage. This would make propulsion less efficient. A possible reason is muscle fatigue at the ankle plantar flexor muscles. Such hypothesis would be consistent with previous investigations, reporting that such muscle group is particularly stressed, during trail running, showing both reduced central activation and strength loss [25]. With respect to the knee joint, no clear relationship has been found between kinematics and kinetics [24]. Nonetheless, lower ROM during propulsion phase (i.e., knee extension) suggests possible muscle fatigue at the knee extensors. In this regard, Giandolini et al. [26] investigated strength loss of plantar flexors and knee extensors with respect to exercise duration, considering both level running and trail running data. The authors reported comparable trend between level and trail running, suggesting that strength loss at plantar flexors and knee extensors is mainly related to exercise duration, rather than elevation gain. Based on such findings and considering the duration of the present trail running trial, strength loss at plantar flexors and knee extensors would be ∼ 15%.

Results confirmed the hypothesis of shorter FT in later race stages. Such findings are consistent with previous studies, where a positive relationship between running speed and FT was reported [7]. From a kinetic perspective, FT is determined by the impulse of ground reaction force (GRF) in the normal direction, i.e., the area underneath the normal GRF curve during CT. To maintain the same area (i.e., the same FT) during shorter CT, larger normal GRF are needed, and vice versa. Therefore, the combination of longer CT and shorter FT in our data clearly indicate the production of lower GRF and lower GRF impulse in the normal direction, in the final stage. Such findings corroborate our aforementioned hypothesis of less efficient propulsion phase during stance, in particular at the ankle joint. Provided that during flight phase body behaves like a ballistic object, a shorter FT, in turn results into lower SL. Finally, the combination of similar SF and longer CT explains the higher DF found in the final stage. With respect to upper body, trunk was significantly more tilted forward across the entire gait cycle (see Figure 7A). Forward lean has been reported to occur in presence of fatigue at the paraspinal muscles [8]. Reviewing the effects of acute fatigue in overground level running, it was reported that higher forward lean combined with reduced hip extension during swing phase, as found in the present study, may be a strategy to reduce stress on lower limbs joints [27], since higher trunk forward tilt has been indicated as a strategy to reduce stress at the joints and the risk of injury [28]. Nonetheless, such posture has also been reported as detrimental to running economy [29]. Given the increasing popularity of trail running, future works may investigate whether such findings apply to incline running as well. With respect to trunk axial rotation, no differences were found between race stages (see Figure 7C).

### 4.2 Downhill

With respect to DH section instead, results confirmed the hypothesis of lower SL in the final stage (see Figure 5B). However, such decrease did not result into a lower running speed, as SF values showed a slight increase, in the final stage (see Figure 5A). Similarly to UH sections, it appears that running speed is more strongly regulated by SL rather than SF, as running speed and SL present the same trend. Results confirmed the hypothesis of longer CT in the final stage, despite the effect was not large, from a quantitative standpoint (see Figure 5C). FT, on the other hand, showed an evident decrease in the final stage (see Figure 5C). With respect to initial stage, the decrease in FT was larger than the increase in CT in the final stage, resulting in a lower stride period, which explains the slight tendency to higher SF in the final stage. With respect to kinematics, athletes showed a systematic tendency to higher dorsiflexion at the ankle joint in the final stage, which becomes significant during the propulsion sub-phase of stance (see Figure 6B). This seems to corroborate the hypothesis of muscle fatigue at the ankle plantar flexors. Interestingly, in the final stage, both hip and knee were significantly less flexed in early swing phase and contralateral stance phase, despite peak flexion values during swing did not differ between race stages (see Figures 6D,F). In previous works it was suggested that different kinematics of the swing leg may contribute to change its inertial properties and, ultimately, impact performance. In fact, lower knee flexion “has been shown to hinder the hip flexors from propelling the leg quickly forward due to the associated changes in the moment of inertia of the swing leg” [7, 29]. In trail running, higher hip and knee flexion during swing phase have been reported, when running at higher speed [9]. Next to the above considerations about inertial properties of the swing leg, it was suggested that these kinematic differences may result in a more forward displacement of the COM of the swing leg. Ultimately, this would contribute to maximize the forward momentum of the full body, thus optimizing the propulsion phase of contralateral stance leg. This would result into higher horizontal speed at toe off which, combined with the longer FT, would explain the higher SL in the initial rage stage. When considering the knee joint, the peak extension during the swing phase was lower in the final stage, as observed for UH sections. This finding supports the hypothesis of muscle fatigue at the knee extensors as well. With respect to upper body, trunk axial rotation between race stages was different approximately at 10-20% and 60-70% of the gait cycle (see Figure 7D). These ranges coincide with those where the contralateral leg is in the swing phase and its hip flexion differs between race stages. In particular, during initial stage athletes expressed higher hip flexion of the (contralateral) swing leg combined with higher trunk ipsilateral rotation, whilst the opposite is true in the final stage. Most likely, this is a strategy to maintain null the full body angular momentum about the vertical axis, with upper body and lower body rotating in opposite directions. If a mismatch occurred, it would result in a non straight running trajectory. These results are consistent with those reported by Genitrini et al. [9], where athletes running at lower speeds expressed lower hip and knee joint flexion during swing, as well as lower ROM for trunk axial rotation. With respect to trunk lean, no differences were found between race stages (see Figure 7B). In last stance, longer CT, shorter FT and similar SF resulted into a higher duty factor in the final stage.

### 4.3 Summary and limitations

The present study demonstrates how, comparing UH and DH sections, almost identical changes in performance-related variables (STP) can be elicited by dissimilar changes in technique-related variables (joint angle kinematics). This information is of relevance for sportspeople, given the increasing popularity of wearable devices to monitor training and performance. In fact, sensors such as foot pods or similar usually provide running metrics such as speed and STP, but no information about kinematics. Considering incline running, when observing a similar relationship between speed and STP in both UH and DH conditions, users would likely assume that this are caused by as much similar changes in running technique. The present work demonstrates that this is not the case. In particular, when comparing race stages, ankle joint kinematics differs in both stance and swing phases in UH sections, but in the stance phase only in DH sections. Knee joint kinematics differs in both stance and swing phases in UH sections, but in swing phase only in DH sections. Between race stages and with respect to upper body, in UH sections differences were found for the trunk in the sagittal plane, whilst in DH sections differences were found in the transverse plane. Therefore, sportspeople may target specific and different aspects of running technique, when running on different inclines.

A limitation of the present study is represented by the walking gait cycles: a small part of the decrease in performance in the final stage is explained by the fact that subjects were walking instead of running, and not uniquely by the differences in running biomechanics. Nonetheless, we believe that the criteria of excluding those subjects for whom the number walking gait cycles was dissimilar (i.e., more than 10%) between race stage may offset such limitation. Also, the population tested in the present study consists of amateur athletes, therefore, it is possible that the present biomechanical adaptations differ from those occurring in elite trail runners. Moreover, in trail running, psychological factors such as self motivation and willingness to take risks on technical terrain play a critical role. Therefore, future investigations may combine biomechanical tests with questionnaires focusing on the mental condition of the athletes. In last stance, it is well established that wearable sensors, usable in the field, do not provide the same accuracy as gold standard methods in laboratory conditions. To limit as much as possible this aspect, sensors were carefully fixed based on the instructions of the manufacturer. Also, as sensors may move during the test, we calibrated both before and after the test, so to have a *a posteriori* calibration, in case of low data quality due to small movements of the sensors with respect to the beginning of the test.

## 5 Conclusion

This study showed the adaptations occurring in technique-related variables, in the final stage of a trail running field test. Also, it was shown such adaptations impact performance-related variables and, therefore, running speed. In general, the final (fatigued) stage was associated to a more crouched running style.

Furthermore, kinematic changes between initial and final stage were different between UH and DH sections, confirming the situation-specificity of adaptations in technique-related variables.

With respect to performance-related variables, on the other hand, changes in STP are in line with previous studies where faster and slower trail runners were compared [9]. Also, changes in STP were almost identical in UH and DH sections, confirming that their trend largely depends on running speed, rather than other boundary conditions.

Such findings are relevant to coaches and practitioners, informing that different kinematic adaptations occur on different slopes, despite similar trends in STP are observed. This is particularly important considering the increasing popularity of wearable technology for training/performance monitoring. In fact, most of the commercially available foot pods provide information about STP and other relevant metrics, but not about kinematics. Therefore, sportspeople may believe that similar changes in STP are elicited by similar changes in their movement pattern, whilst the findings of the present study demonstrate that this is not the case. In late race stages, most likely due to the onset of acute fatigue, trail runners and coaches may want to focus on (different) specific aspects of running technique, when running on different slopes. For instance, in UH sections it may be advisable to limit ankle dorsiflexion during stance phase and forward trunk lean, whilst in DH sections it could be beneficial to maintain high knee and hip flexion during swing phase, which may contribute to optimize propulsion in forward direction during contralateral stance phase.

## Data Availability

The raw data supporting the conclusions of this article will be made available by the authors, without undue reservation.
